# Repellent Activity of DEET Combined with Botanical Compounds Against *Amblyomma sculptum* Nymphs: Laboratory and Field Evaluations

**DOI:** 10.3390/pathogens14050495

**Published:** 2025-05-17

**Authors:** Mayara Macêdo Barrozo, Haile Dean Figueiredo Chagas, Gabrielly Bernardes Rodrigues Damaceno, Emilly Faria Santos, Rafael Assunção Carvalho, Isabela Santos Silva, Francisca Letícia Vale, Lainny Jordana Martins Pereira e Sousa, Hermes Ribeiro Luz, Lorena Lopes Ferreira, Caio Monteiro

**Affiliations:** 1 Centro de Parasitologia Veterinária, Escola de Veterinária e Zootecnica, Universidade Federal de Goiás, Av. Esperança, s/n—Campus Samambaia, Goiânia 74690-900, GO, Brazil or mayarabarrozo@discente.ufg.br (M.M.B.); haile.dean@discente.ufg.br (H.D.F.C.); emillyfaria@discente.ufg.br (E.F.S.); rafaelassuncao@discente.ufg.br (R.A.C.); isabelasantoscbio@gmail.com (I.S.S.); leticiadetecta@gmail.com (F.L.V.); lainnyjordana@gmail.com (L.J.M.P.e.S.); 2 Laboratory of Nanosystems and Drug Delivery Devices (NanoSYS), School of Pharmacy, Universidade Federal de Goiás, Rua 240, Setor Leste Universitário, Goiânia 74605-170, GO, Brazil; gabriellybrd@hotmail.com; 3 Departamento de Patologia, Programa de Pós-Graduação em Biotecnologia do Renorbio, Ponto Focal Maranhão, Universidade Federal do Maranhão, São Luís 65085-580, MA, Brazil; hermes.luz@ufma.br; 4 Departamento de Medicina Veterinária Preventiva, Escola de Veterinária, Universidade Federal de Minas Gerais, Av. Antônio Carlos 6627 Caixa Postal 567, Campus Pampulha, Belo Horizonte 31270-901, MG, Brazil; loren4_lopes@hotmail.com; 5 Instituto de Patologia Tropical e Saúde Pública, Universidade Federal de Goiás, Rua 235 s/n, Setor Universitário, Goiânia 74605-050, GO, Brazil

**Keywords:** *Amblyomma cajennense* complex, botanical products, essential oils, eugenol, methyl eugenol, 1,8-cineole

## Abstract

This study evaluated the repellent activity of DEET, eugenol, methyl eugenol, 1,8-cineole, and their combinations against *Amblyomma sculptum* nymphs through laboratory and field tests. Initially, the botanical compounds were tested individually in Petri dish assays to assess repellent activity over various intervals (1 min to 168 h) at concentrations of 2%. Eugenol and methyl eugenol exhibited the highest average repellent percentages (60 to 90%), with significant effects (*p* < 0.05) across most evaluation periods, in contrast to 1,8-cineole. Therefore, eugenol and methyl eugenol were selected for combination testing with DEET. The combinations of 7% DEET + 1% eugenol and 7% DEET + 2% methyl eugenol showed the best results, with average repellent efficacy exceeding 85%. The first combination was selected for field evaluation. In this test, volunteers wore treated socks and walked for two hours in a naturally infested area. A significant reduction (*p* < 0.05) in the number of ticks recovered from the socks was observed in the 7% DEET and 7% DEET + 1% eugenol treatments, with efficacies of 82.5% and 88.5%, respectively, after 120 min. Isolated eugenol showed limited field efficacy, with significant repellent activity (*p* < 0.05) lasting only 20 min. The results highlight the potential of eugenol and methyl eugenol as repellent compounds, especially when combined with DEET. The field study confirmed the efficacy of the 7% DEET + 1% eugenol.

## 1. Introduction

*Amblyomma sculptum* is one of the most widely distributed tick species in South America [1,2]. While its primary hosts include equines, capybaras, and tapirs, this tick exhibits low host specificity, allowing it to parasitize a variety of wild and domestic animals, and humans [3,4,5]. It is a known vector of *Rickettsia rickettsii*, the etiological agent of Brazilian spotted fever (BSF), an acute febrile illness with a high mortality rate that can reach 80% if not promptly diagnosed and treated [6,7]. The period of highest disease incidence correlates directly with the peak abundance of *A. sculptum* nymphs in the environment, which occurs between June and October, when the risk of transmission is heightened [8,9].

The use of repellents is the most commonly employed method to prevent tick bites, with DEET (N, N-diethyl-3-methylbenzamide) being the organosynthetic compound considered the gold standard among repellents [10,11,12,13,14]. Additionally, DEET is known to induce both spatial and contact repellency in *A. sculptum* under laboratory and field conditions [14,15,16,17,18]. Despite its proven efficacy and safety, DEET use is restricted in some countries, particularly for children, pregnant women, and the elderly, due to concerns about its potential toxicity [19,20]. In the Brazilian market, commercial formulations containing DEET are available at various concentrations (6–15%) [21]. While increasing DEET concentration may enhance repellent efficacy, it also raises concerns regarding toxicity, especially for vulnerable populations such as pregnant women, children, and the elderly [22,23]. One alternative strategy to improve efficacy while mitigating potential risks is to combine DEET with other repellent compounds in a single formulation.

Research suggests that botanical compounds, when combined with DEET, can enhance its repellent activity and provide increased protection against hematophagous arthropods, including ticks [12,24,25,26,27,28]. In Brazil, commercial products containing DEET combined with citronellal as active ingredients are already available, as well as other botanical compounds commonly found in essential oils (EOs), such as geraniol, linalool, and limonene. However, these additional botanical compounds are often classified as fragrances rather than active ingredients [29]. A recent study evaluating the effectiveness of various commercial repellents against *A. sculptum* nymphs under laboratory and field conditions found that a formulation containing DEET (6.65%) and orange EO, along with other botanical compounds—such as citronellal, geraniol, and linalool—yielded the best results [14]. It is important to note that all of these compounds have been documented in the literature for their repellent activity against ticks [12,30]. Thus, it is hypothesized that these botanical molecules may significantly contribute to the overall repellent effect observed in commercial DEET-based products.

The phenylpropanoids eugenol and methyl eugenol, along with the terpene 1,8-cineole (also known as eucalyptol), are compounds found in essential oils (EOs) that have been extensively documented for their broad range of biological activities. These include antifungal, antimicrobial, anthelmintic, insecticidal, acaricidal, and repellent effects against hematophagous arthropods, particularly ticks [27,31,32,33,34,35,36,37,38,39,40]. These compounds are considered safe for human use and have been widely utilized in the pharmaceutical, food, dental, and other industries [41,42,43]. The objectives of the present study were as follows: (i) to assess the repellent activity of eugenol, methyl eugenol, and 1,8-cineole against *A. sculptum* nymphs, the developmental stage most commonly associated with the transmission of *R. rickettsii* to humans [44]; and (ii) to identify the most promising compounds for further testing in combination with DEET under both laboratory and field conditions. We hypothesize that these binary combinations (DEET with botanical compounds) will enhance repellent efficacy.

## 2. Materials and Methods

### 2.1. Ticks

Engorged *A. sculptum* females were obtained from naturally infested cattle at the School of Veterinary Medicine and Animal Science (EVZ), Federal University of Goiás (UFG), Goiânia, Brazil. These females were incubated at 27 ± 1 °C and >80% relative humidity to allow egg-laying and larval hatching. The resulting *A. sculptum* larvae were fed on rabbits (*Oryctolagus cuniculus*, California × New Zealand breed) to obtain engorged larvae, which were then incubated under the same conditions described above to obtain unfed nymphs. All procedures involving animals were approved by the Animal Use Ethics Committee of UFG (CEUA-UFG 053/21). Unfed nymphs aged between 15 and 60 days were selected for laboratory repellency bioassays, as this developmental stage is primarily associated with the transmission of *R. rickettsii* to humans [44].

### 2.2. Test Compounds

Eugenol, methyl eugenol, 1,8-cineole, and DEET were purchased from Sigma-Aldrich^®^ (St. Louis, MO, USA) at 98% purity. In laboratory bioassays with unfed *A. sculptum* nymphs, eugenol, methyl eugenol, and 1,8-cineole were individually tested at a concentration of 2%. In combination tests, DEET was used at a concentration of 7%, while botanical compounds (eugenol and methyl eugenol) were tested at concentrations of 1% and 2%. Ethanol (Neon^®^-99,5°GL), at a concentration of 50% (*v*/*v*), was used as a solvent to prepare the test solutions for both laboratory and field assays, as previous studies have demonstrated its low toxicity to unfed stages of *A. sculptum* [45,46]. The concentrations and combinations yielding the best results in laboratory tests were selected for subsequent field evaluations.

### 2.3. Laboratory Bioassays (Petri Dish Assay—One Choice)

#### 2.3.1. Eugenol, Methyl Eugenol, and 1,8-Cineole

**Figure 1:**
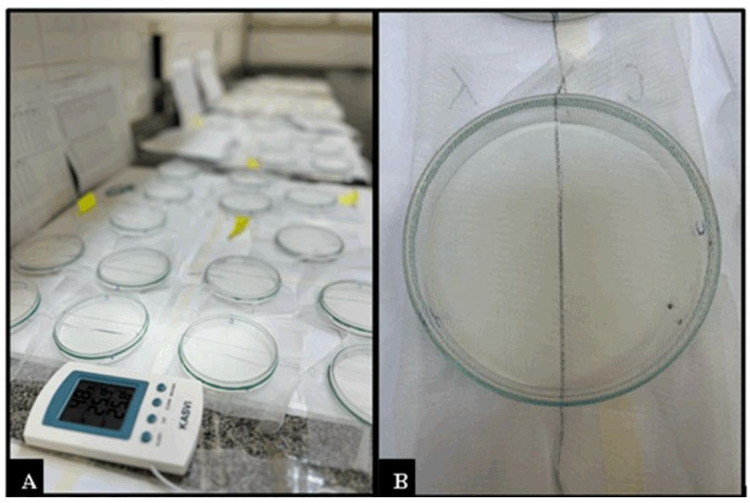
Laboratory bioassay for evaluating the repellency of botanical compounds (eugenol, methyl-eugenol, and 1,8-cineole) against *Amblyomma sculptum* nymphs. (**A**) Setup of laboratory bioassays using Petri dishes to assess contact repellency under controlled temperature and humidity conditions. (**B**) Detail of a 10 cm Petri dish containing two semicircles of filter paper, covered with a polyamide fabric to prevent tick escape and simulate interaction with the environment.

The botanical compounds eugenol, methyl eugenol, and 1,8-cineole were tested at a concentration of 2% using a Petri dish bioassay, following the methodology proposed by Bissinger et al. [47] and adapted from Barrozo et al. [46]. The experiments were conducted in an air-conditioned room under controlled conditions (27 ± 1 °C and 70 ± 10% relative humidity). For the assay, glass Petri dishes (10 cm in diameter) were used, each containing two semicircles (31.8 cm^2^ in area) of filter paper (Whatman, Maidstone, UK, cat. no. 1001), with each semicircle covering half of the bottom of the plate (Figure 1A,B).

Glass Petri dishes (10 cm in diameter) were used for the assays. Each dish contained two semicircles of filter paper (31.8 cm^2^ each; Whatman, Maidstone, UK, cat. no. 1001) positioned to evenly cover the bottom surface. One semicircle was treated with 150 μL of the test compound, while the other received 150 μL of 50% ethanol (solvent control). After application, the papers were allowed to dry for 10 min in a fume hood (Permution) before being placed into the dishes. Six unfed *A. sculptum* nymphs were introduced at the central line where the treated and control semicircles met. To prevent escape, each Petri dish was covered with a thin transparent fabric made of 100% polyamide and secured with an elastic band. Additionally, 150 μL of the same test solution was applied to one half of the fabric (corresponding to the side above the treated semicircle) and allowed to dry for 10 min in the fume hood.

Two control groups were established to evaluate the potential influence of the solvent and filter paper on tick behavior. In the first control group, one semicircle was treated with 50% ethanol (solvent), while the other remained untreated (ethanol-treated paper vs. untreated paper). In the second control group, neither semicircle was treated (blank control: untreated paper vs. untreated paper). These controls were included to confirm that neither the filter paper nor the solvent affected tick positioning, thereby ensuring that any repellency observed in the experimental groups could be attributed solely to the presence of the botanical compounds.

The position of the ticks in each Petri dish was recorded at 1, 15, and 30 min, and at 1, 2, 3, 4, 24, 48, 72, 96, and 168 h after the start of the experiment. Ticks found on the untreated semicircle were considered repelled. The mean repellency percentage was calculated based on the proportion of ticks located on the control (untreated) side of the dish. Experiments were conducted on two separate days, using five dishes per day (each dish representing one experimental unit), totaling ten replicates. Botanical compounds that achieved a mean repellency of ≥75% were selected for further testing in binary combinations with DEET.

#### 2.3.2. Eugenol, Methyl Eugenol, and Their Binary Combinations with DEET

The same methodology described in Section 2.3.1 was applied to the following treatments: 1% eugenol; 2% eugenol; 7% DEET; 7% DEET + 1% eugenol; 7% DEET + 2% eugenol; 1% methyl eugenol; 2% methyl eugenol; 7% DEET + 1% methyl eugenol; and 7% DEET + 2% methyl eugenol. Two control groups were also included, as described in the previous section. The most effective binary combination, based on repellency results, was selected for subsequent evaluation in the field bioassay.

### 2.4. Field Bioassays (Sock Assay)

**Figure 2:**
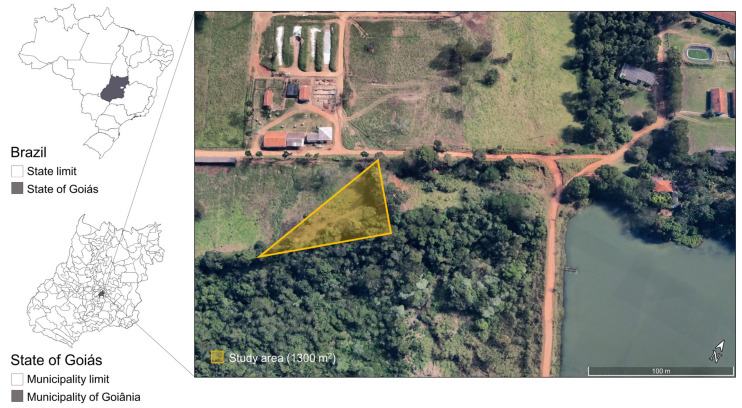
Geographical region in Brazil where the field assays were conducted. The triangle indicates the area where the volunteers walked wearing treated (7% DEET, 1% eugenol, and 7% DEET + 1% eugenol) or untreated clothing to evaluate the repellency of the compounds against *Amblyomma sculptum* nymphs. The area consists of *Panicum maximum* pastures inhabited by capybaras and cattle and is naturally infested with *A. sculptum*.

Field bioassays were conducted with the participation of 10 volunteers (7 women and 3 men), who rotated randomly on the test days. All participants reported no known allergies or sensitivities to the tested compounds. This study was approved by the Research Ethics Committee (CEP/UFG—No. 4.955.565). Field repellency tests were carried out at the experimental farm of the School of Veterinary Medicine and Animal Science (EVZ/UFG), in Goiânia, Goiás, Brazil (16°35′39.6″ S, 49°16′58.8″ W) (Figure 2). The area consists of *Panicum maximum* pastures inhabited by capybaras and cattle and is naturally infested with *A. sculptum* [48,49]. Notably, this site has a confirmed history of Brazilian spotted fever (BSF) cases and is characterized by a high density of *A. sculptum*. Over a two-year survey period, 100,627 larvae, 10,055 nymphs, and 6,977 adult ticks were collected [48].

The methodology was adapted from Bissinger et al. [50]. Field trials were conducted on five separate days between July and August 2021, a period during which *A. sculptum* nymphs were predominant in the pastures [8,48]. On each study day, tests were performed in duplicate for each treatment between 9:00 and 11:30 a.m., resulting in a total of 10 replicates per treatment. The testing period was selected based on two criteria, as follows: (i) the collection timeframe used in a previous study that documented the seasonal dynamics of *A. sculptum* in the area [48], and (ii) the peak nymph activity in the field, as observed by the research team.

**Figure 3:**
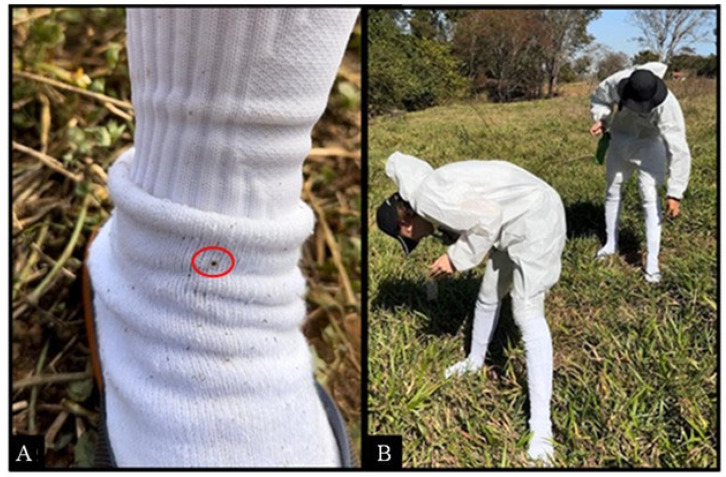
Field trials (sock assay) to evaluate the repellency of different compounds containing 7% DEET, 1% eugenol, and 7% DEET + 1% eugenol against *Amblyomma sculptum* nymphs. (**A**) Tick climbing on the white sock used in the field tests, highlighted with a red circle. (**B**) Volunteers wearing protective coveralls and white socks during the field trials, conducted in an area naturally infested with *A. sculptum*.

The volunteers wore appropriate personal protective equipment (PPE), including white coveralls with hoods. The sleeve ends, zippers, and buttons were sealed with adhesive tape. The participants also wore Kanxa^®^ brand socks (composition: 49% polyamide, 34% cotton, 11% polyester, and 6% elastodiene) over the coveralls, extending up to the knee region (Figure 3A,B). Prior to spraying, the external surface area of each sock was measured to determine the appropriate volume of solution to be applied (1 mL per 600 cm^2^).

The solutions containing the repellent compounds were applied to the socks 15 min prior to the start of the test using a spray bottle, ensuring complete coverage of the area between the knee and ankle. Each sock was used only once. The volunteers wore a sock treated with the test solution on one leg and an identical sock treated with 50% ethanol (negative control) on the other. The volunteers were instructed to walk randomly for 20 min during each evaluation period over an area of approximately 80 m^2^, at a slow pace (approximately 30 steps per minute) (Figure 3A,B). During the walk, the participants were instructed to collect any ticks that climbed onto the socks.

The walk took place primarily along the edge of the forest, a region with the highest incidence of *A. sculptum*. The total duration of each test was 120 min. Ticks were collected at 20, 40, 60, and 120 min using transparent adhesive tape. Collected ticks were affixed to white paper with additional adhesive tape and stored for later counting. Only nymphs were quantified, and identification followed the methods described by [51,52]. The following formula was used to calculate repellency:

Repellency (%) = (C − T)/C × 100

where

C = number of nymphs collected from the control sock;

T = number of nymphs collected from the treated sock.

The compounds tested in the field assay were as follows: 50% ethanol (negative control); 1% eugenol; 7% DEET (positive control); and 7% DEET + 1% eugenol. These treatments were selected based on their repellency performance in the two preceding laboratory bioassays.

### 2.5. Data Analysis

Repellency results were expressed as mean ± standard deviation (SD). The repellency percentage for each compound and concentration was calculated based on the number of ticks that remained on the control side of the Petri dish, or on the sock with or without treatment. The chi-square test was used to compare tick distribution, adopting a significance level of *p* < 0.05. Statistical differences in field data were assessed using one-way ANOVA or Student’s *t*-test, with analyses performed in GraphPad Prism version 5.03 (GraphPad Software, Inc., La Jolla, CA, USA).

## 3. Results

### 3.1. Laboratory Assays

#### Eugenol, Methyl Eugenol, and 1,8-Cineole

In the control groups (50% ethanol and blank), no significant difference (*p* > 0.05) in tick distribution was observed across most time points, except at 48, 72, and 168 h (Table 1). Among the treated groups, the highest repellency percentages were recorded for 2% eugenol and 2% methyl eugenol. For 2% eugenol, the repellency values ranged from 60% to 90%, with a mean of 75.5%. Methyl eugenol at 2% showed repellency ranging from 55% to 88.3%, with a mean of 78.3%. Both botanical compounds showed statistically significant differences in tick distribution (*p* < 0.05) at nearly all evaluation time points, except at 72 and 96 h for eugenol, and at 96 and 168 h for methyl eugenol (Table 1).

In contrast, 2% 1,8-cineole exhibited repellency values ranging from 51.7% to 76.7%. Statistically significant differences (*p* < 0.05) in tick distribution were observed at only three time points: 30 min, 3 h, and 48 h (Table 1). At the end of all evaluation periods, the mean repellency values for 2% eugenol, 2% methyl eugenol, and 2% 1,8-cineole were 75.5%, 78.3%, and 58.8%, respectively. Based on these findings, eugenol and methyl eugenol were selected for further evaluation in binary combinations with DEET.

### 3.2. Binary Combinations of Eugenol, Methyl Eugenol, and DEET

No significant repellency was observed in either control group (50% ethanol or blank), as indicated by the absence of significant differences in tick distribution (*p* > 0.05) (Table 2 and Table 3). For treatments with methyl eugenol, repellency ranged from 51.7% to 93.3% at the 1% concentration, and from 55.0% to 96.7% at 2%. Statistically significant differences in tick distribution (*p* < 0.05) were observed at nearly all evaluation time points, except for two for each concentration (1%: 96 and 168 h; 2%: 72 and 168 h). In contrast, 7% DEET showed significant repellency (*p* < 0.05) only up to the 24 h time point, with repellency values ranging from 43.3% to 91.7% (Table 2).

Importantly, the binary combinations of 7% DEET with methyl eugenol (1% or 2%) resulted in significant repellency across all evaluation time points (*p* < 0.05). The repellency values for the 7% DEET + 1% methyl eugenol combination ranged from 66.7% to 96.7%, while the 7% DEET + 2% methyl eugenol combination achieved repellency between 63.3% and 100% (Table 2). At the end of all evaluation periods, the mean repellency values for 1% methyl eugenol, 2% methyl eugenol, 7% DEET, 7% DEET + 1% methyl eugenol, and 7% DEET + 2% methyl eugenol were 77.5%, 78.9%, 74.1%, 82.9%, and 85.4%, respectively (Table 2).

For treatments with 1% eugenol, significant repellency (*p* < 0.05) was observed at nearly all evaluation time points, except at 96 h, with efficacy ranging from 62.2% to 93.3%. Similarly, 2% eugenol resulted in significant repellency (*p* < 0.05) across most time points (58.3% to 93.3%), except at 96 h. In contrast, 7% DEET demonstrated significant repellency only up to 24 h (Table 3). All binary combinations of DEET and eugenol produced significant repellency (*p* < 0.05) against *A. sculptum* nymphs for up to 168 h. Efficacy ranged from 78.3% to 98.3% for the 7% DEET + 1% eugenol combination and from 76.7% to 93.3% for the 7% DEET + 2% eugenol treatment.

At the conclusion of the evaluation period, the mean repellency values for 1% eugenol, 2% eugenol, 7% DEET, 7% DEET + 1% eugenol, and 7% DEET + 2% eugenol were 77.6%, 82.9%, 74.5%, 88.1%, and 85.5%, respectively (Table 3).

Based on these results, all combinations of DEET with the botanical compounds eugenol and methyl eugenol (at both 1% and 2%) resulted in statistically significant differences (*p* < 0.05) in tick distribution at all time points, with mean efficacy values exceeding 80%. These findings indicate that all combinations demonstrated comparable repellent potential. Consequently, the 7% DEET + 1% eugenol formulation was selected for field testing, as it achieved the highest mean repellency (88.1%).

### 3.3. Field Bioassays

**Figure 4:**
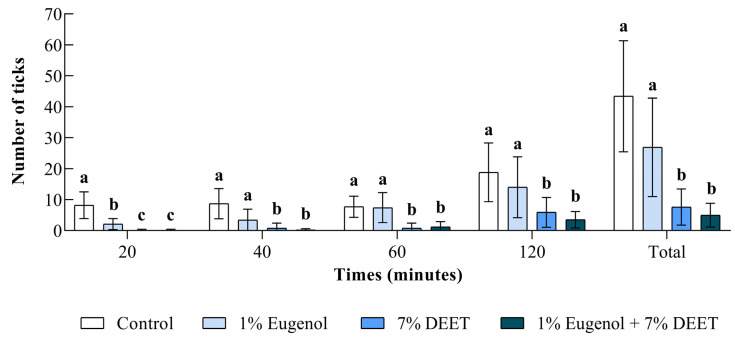
Number of *Amblyomma sculptum* nymphs collected while climbing on socks treated with 1% eugenol, 7% DEET, or a combination of 7% DEET + 1% eugenol, and on untreated socks (control: 50% ethanol), at different evaluation times (20, 40, 60, and 120 min), during a field trial in a naturally infested area. Bars represent the mean ± standard deviation. Different letters indicate statistically significant differences between treatments at the same evaluation time (ANOVA, *p* < 0.05).

The number of ticks collected in each treatment group is shown in Figure 4. All nymphs collected during the field assays were identified as *A. sculptum*. After 20 min, the number of nymphs collected from socks treated with 1% eugenol, 7% DEET, and the 7% DEET + 1% eugenol combination was significantly lower (*p* < 0.05) than that collected from the control group. From the 40 min time point onward, the number of ticks collected from socks treated with 7% DEET or 7% DEET + 1% eugenol remained significantly lower (*p* < 0.05) compared to both the control and 1% eugenol groups. At the end of the 2 h evaluation period, only 7.6 and 5.1 nymphs were collected from the 7% DEET and 7% DEET + 1% eugenol groups, respectively, with no significant difference between these two treatments (*p* > 0.05). In contrast, 43.4 and 29.9 nymphs were collected from the control and 1% eugenol groups, respectively. In the cumulative assessment at 2 h, no significant difference was observed between the control and 1% eugenol groups (*p* > 0.05); however, both showed significantly higher tick counts (*p* < 0.05) compared to the 7% DEET and 7% DEET + 1% eugenol treatments (Figure 4).

**Figure 5:**
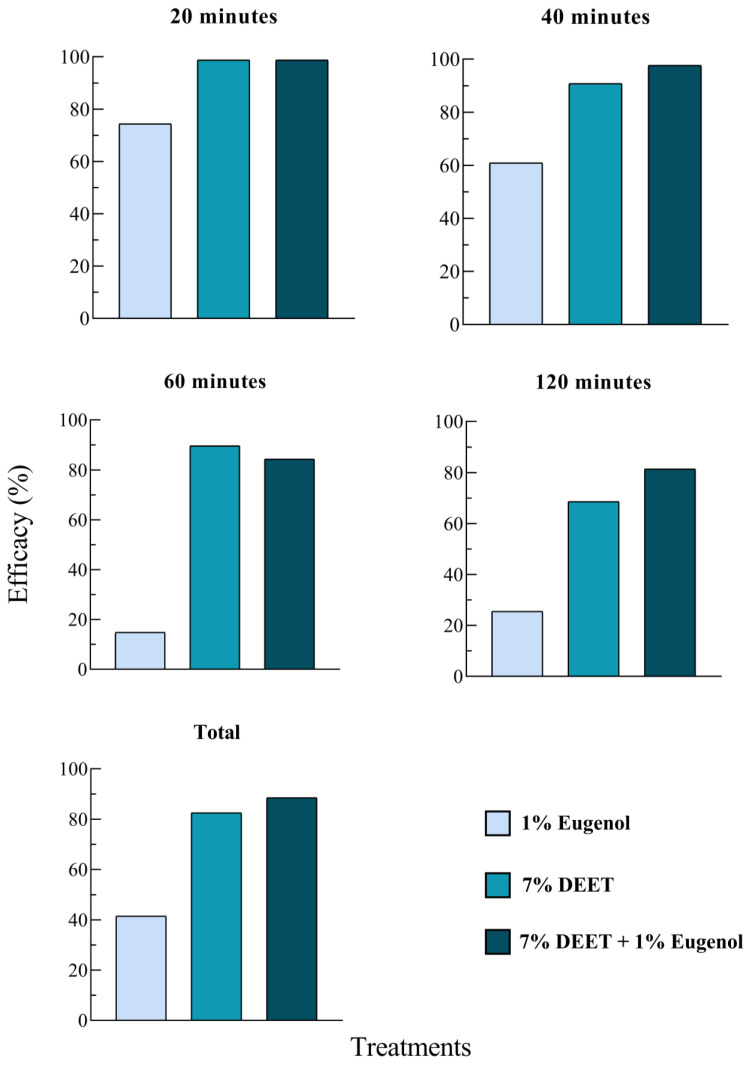
Repellent efficacy (%) observed in field trials (sock assay) using 1% eugenol, 7% DEET, and the combination of 7% DEET + 1% eugenol against unfed *Amblyomma sculptum* nymphs at different evaluation times (20, 40, 60, and 120 min) in a naturally infested area.

Treatment efficacy was also evaluated and is presented in Figure 5. Eugenol at 1% showed repellency of approximately 70% and 60% at 20 and 40 min, respectively. However, at 60 and 120 min, repellency declined markedly, dropping to around 20%. In contrast, 7% DEET maintained repellency above 80% at 20, 40, and 60 min. The 7% DEET + 1% eugenol combination demonstrated repellency greater than 80% at all evaluation time points (Figure 5). Considering the total number of ticks collected over the 2 h evaluation period, the 7% DEET + 1% eugenol combination exhibited the highest efficacy, with 88.5% repellency, followed by 7% DEET alone (82.5%) and 1% eugenol (41.5%) (Figure 5).

## 4. Discussion

This study is the first to report the repellent activity of binary combinations of DEET with botanical compounds against *A. sculptum* nymphs under both laboratory and field conditions. We also present novel data on the repellency of eugenol and DEET, individually and in combination, for protecting humans against nymphal bites of this tick species under natural field conditions, when applied to clothing.

In the first laboratory experiment, the compounds eugenol, methyl eugenol, and 1,8-cineole, at 2%, exhibited repellent activity against *A. sculptum* nymphs, with eugenol and methyl eugenol showing the highest efficacy (average repellency > 75% after 168 h). Previous studies have demonstrated the repellent activity of eugenol against several tick species, including *Rhipicephalus microplus*, *Dermacentor variabilis*, *Ixodes scapularis*, *Ixodes ricinus*, and *Hyalomma scupense* [35,39,53,54]. Although no repellent data are available for *A. sculptum*, this phenylpropanoid has demonstrated acaricidal activity against this species [55,56]. Eugenol has also shown repellent activity against other arthropods, including *Aedes aegypti* [57], *Anopheles stephensi* [58], triatomines [59], beetles [60], and ants [61], highlighting its potential in repellent development. Regarding methyl eugenol, repellent activity has been documented against other arthropods, including mosquitoes and ants [40,62]; however, to our knowledge, this is the first study to demonstrate its repellent effect against ticks. Its acaricidal activity against *R. microplus* has been previously reported [63,64].

In the current study, 2% 1,8-cineole demonstrated lower repellent activity (58% at 168 h) compared to eugenol and methyl eugenol at the same concentration. In contrast, previous studies—such as that by İnceboz et al. [65]—have reported more promising results, with repellency lasting up to 72 h against *Hyalomma marginatum* in both in vitro and in vivo experiments, showing superior efficacy compared to DEET. These discrepancies may be attributed to the (i) variation in tick species and (ii) the use of 1,8-cineole encapsulated in β-cyclodextrin (β-CD), as shown in the study by İnceboz et al. [65], which likely enhanced the compound’s stability and repellent efficacy. The literature suggests that encapsulation can improve the release profile, stability, and overall effectiveness of botanical compounds [66,67]. Therefore, this strategy—potentially applicable not only to 1,8-cineole, but also to eugenol and methyl eugenol—warrants further investigation in future studies targeting *A. sculptum*.

The comparison of repellent activity among the three compounds tested in our study aligns with the findings of Caballero-Gallardo et al. [68], who assessed the repellent properties of essential oils (EOs) from eight plant species against the red flour beetle, *Tribolium castaneum*, a common pest of stored grains. The authors observed that EOs from *Ocimum campechianum* (Oc) and *Piper divaricatum* (Pd), both rich in methyl eugenol (Oc = 12%, Pd = 33%), were more effective than the EO from *Hedychium coronarium* (Hc), which had 1,8-cineole as the predominant compound (Hc = 31%). In our laboratory experiments, we found that both eugenol and methyl eugenol, even at relatively low concentrations (1% and 2%), exhibited repellent activity and residual effects comparable to 7% DEET. These findings are consistent with those of He et al. [40], who conducted multiple assays to evaluate the repellent activity of eugenol and its derivatives against ants, using DEET as a positive control. In all three repellency assays, eugenol, methyl eugenol, and DEET demonstrated similar efficacy, with no significant differences observed among the treatments [40].

In the laboratory studies, the combinations of DEET with eugenol and DEET with methyl eugenol demonstrated statistically significant differences in tick distribution across all evaluation periods, with efficacy exceeding 80%. This effect was not observed when the compounds were tested individually. These findings support our hypothesis that combining botanical compounds with DEET can enhance its repellent efficacy. This hypothesis is based on the following three key points: (i) the presence of botanical compounds in commercial repellents containing DEET in Brazil, where they are classified as flavoring agents but are likely to contribute to the product’s efficacy [14,29]; (ii) previous reports showing improved efficacy of chemical acaricides when combined with botanical compounds [69,70,71,72]; and (iii) the hypothesis that mixtures of DEET with compounds acting on the cholinergic system may enhance repellent activity [73], along with evidence that eugenol inhibits acetylcholinesterase activity in ticks [39].

As mentioned in the previous paragraph, all combinations resulted in superior repellency compared to the individual compounds, with statistically significant differences in tick distribution across all evaluation periods. Interestingly, the combinations of 7% DEET with either 1% or 2% eugenol yielded similar results, suggesting a non-dose-dependent effect at least at the doses tested, in the laboratory assay. Burtis et al. [74], in their evaluation of 11 essential oils as tick repellents, also concluded that the most effective formulations did not necessarily contain the highest concentrations of essential oils, supporting the hypothesis of a non-dose-dependent effect. Therefore, due to the challenges of conducting a field study with multiple treatments (such as cost, limited area, and number of volunteers), we selected the combination of 7% DEET + 1% eugenol, as it exhibited the highest efficacy (88% repellent activity) in the laboratory. However, under field conditions—where volatilization of active ingredients is more pronounced—increasing the concentration of botanical compounds may enhance efficacy, a point that warrants further investigation in future studies (for example 7% DEET + 2% eugenol or metil eugenol). 

In the field evaluation, eugenol demonstrated lower efficacy compared to DEET. The repellent efficacy of 1% eugenol was 70% and 60% at 20 and 40 min, respectively, but dropped to approximately 20% at 60 and 120 min, resulting in an overall efficacy of 41%. In contrast, 7% DEET maintained efficacy above 80% during the first three evaluation periods, with an overall efficacy of 82.5%. These results were expected, as DEET is considered the gold standard among repellents and is widely used for preventing bites from hematophagous arthropods, particularly mosquitoes [75,76]. Currently, most repellents available for human use in the Brazilian market contain DEET [77]. A recent study funded by the Ministry of Health and the Pan American Health Organization (PAHO) validated the effectiveness of commercial DEET-based repellents against *A. sculptum* nymph bites in laboratory and field conditions [14].

The reduced efficacy of eugenol under field conditions may be attributed to its high volatility. Eugenol is a phenylpropanoid present in the essential oils of various aromatic plants, and volatility is one of its key physicochemical properties. Unlike laboratory assays, which are performed under controlled conditions, field environments are subject to factors such as solar radiation and elevated temperatures, which can accelerate volatilization and reduce efficacy over time [66,78]. The application of nanotechnology to develop polymer-based formulations that provide controlled release and enhanced stability of eugenol may represent a promising strategy to improve its field performance. Nanoencapsulation has been shown to protect active compounds from degradation, enhance repellent and insecticidal efficacy, and reduce toxicity to non-target organisms [66,67,79].

In the treatment with 7% DEET + 1% eugenol, although no statistically significant differences were observed compared to the group treated with 7% DEET alone, it is noteworthy that repellent efficacy was higher (>80% at all evaluation time points), with a total efficacy of 88%. While the increase in efficacy is modest, this result highlights the potential for future studies aimed at improving repellent performance by carrying out the following: (i) increasing eugenol concentrations in field trials (for example 7% DEET + 2% eugenol); and (ii) developing formulations that encapsulate active ingredients to reduce volatility and enhance both efficacy and safety when applied to clothing or exposed skin [66,67,79,80].

## 5. Conclusions

Eugenol and methyl eugenol exhibited greater repellent activity than 1,8-cineole, with all compounds tested at 2%. In laboratory assays, all binary combinations outperformed the individual compounds. The combination of 7% DEET + 1% eugenol yielded the highest mean repellency at the end of the evaluation period and was, therefore, selected for field testing. In the field study, eugenol alone was effective only during the first 20 min, while 7% DEET and the 7% DEET + 1% eugenol combination maintained efficacy throughout the entire 2 h evaluation period.

## Figures and Tables

**Table 1:** Mean percentage (± standard deviation) of repellent activity observed in unfed *Amblyomma sculptum* nymphs exposed to the botanical compounds eugenol, methyl eugenol, and 1,8-cineole, each at a concentration of 2%, in a Petri dish assay conducted under laboratory conditions (27 ± 1 °C and 70 ± 10% relative humidity).

Evaluation Time		Control (Ethanol)	Control (Blank)	2% Eugenol	2% Methyl Eugenol	2% 1,8-Cineole
Minutes	1	56.7 ± 27.4	53.3 ± 19.0	70.0 * ± 20.4	73.3 * ± 18.3	53.3 ± 30.3
	15	50.0 ± 7.5	45.0 ± 11.8	81.7 * ± 13.9	85.0 * ± 21.7	56.7 ± 34.2
	30	50.0 ± 13.9	40.0 ± 19.0	75.0 * ± 11.8	83.3 * ± 0.0	65.0 * ± 27.4
Hours	1	45.0 ± 20.4	50.0 ± 13.9	90.0 * ± 9.1	76.7 * ± 14.9	51.7 ± 27.9
	2	46.7 ± 21.7	33.3 ± 23.6	86.7 * ± 14.9	73.3 * ± 13.9	55.0 ± 19.0
	3	50.0 ± 14.9	38.3 ± 23.6	88.3 * ± 9.1	88.3 * ± 7.5	76.7 * ± 19.0
	4	51.7 ± 14.9	41.7 ± 16.7	83.3 * ± 0.0	70.0 * ± 18.3	61.7 ± 19.0
	24	50.0 ± 14.9	51.7 ± 11.8	73.3 * ± 14.9	85.0 * ± 14.9	56.7 ± 14.9
	48	55.0 ± 7.5	63.3 * ± 19.0	66.7 * ± 27.4	75.0 * ± 21.7	65.0 * ± 16.7
	72	41.7 ± 25.3	63.3 * ± 19.0	60.0 ± 19.0	73.3 * ± 18.3	55.0 ± 22.4
	96	55.0 ± 30.3	50.0 ± 13.9	61.7 ± 25.3	55.0 ± 21.4	53.3 ± 21.7
	168	48.3 ± 30.3	70.0 * ± 14.9	71.7 * ± 23.6	61.7 ± 16.7	55.0 ± 29.8
Mean repellency (%) ± standard deviation		50.0 ± 4.5	50.0 ± 8.0	75.7 ± 8.2	78.3 ± 9.8	58.8 ± 5.2

* Repellency was considered significant when the number of ticks on the untreated side of the Petri dish was higher than that observed on the treated side, with a statistically significant difference at the 5% level, according to the chi-square test. Control (ethanol): Petri dish containing one semicircle of filter paper treated with 50% ethanol and one untreated semicircle. Control (blank): Petri dish containing two semicircles of untreated filter paper, with no compounds applied.

**Table 2:** Repellency percentage (mean ± standard deviation) of unfed *Amblyomma sculptum* nymphs exposed to 7% DEET and methyl eugenol (1% and 2%), either alone or in combination, in a Petri dish assay conducted under laboratory conditions (27 ± 1 °C and 70 ± 10% relative humidity).

Evaluation Time		Control (Ethanol)	Control (Blank)	1% Methyl Eugenol	2% Methyl Eugenol	7% Deet	7% Deet + 1% Methyl Eugenol	7% Deet + 2% Methyl Eugenol
Minutes	1	56.7 ± 22.5	51.7 ± 22.8	78.3 * ± 17.7	86.7 * ± 15.3	83.3 * ± 17.6	96.7 * ± 7.0	100.0 * ± 0.0
	15	51.7 ± 16.6	41.7 ± 18.0	83.3 * ± 15.7	91.7 * ± 8.8	85.0 * ± 18.3	93.3 * ± 8.6	100.0 * ± 0.0
	30	48.3 ± 21.4	40.0 ± 22.5	85.0 * ± 14.6	91.7 * ± 8.8	91.7 * ± 14.2	96.7 * ± 7.0	96.7 * ± 10.5
Hours	1	48.3 ± 20.0	53.3 ± 15.3	85.0 * ± 16.6	96.7 * ± 7.0	91.7 * ± 11.8	90.0 * ± 17.9	100.0 * ± 0.0
	2	56.7 ± 19.6	41.7 ± 21.2	88.3 * ± 13.7	71.7 * ± 20.9	89.7 * ± 14.3	90.0 * ± 17.9	95.0 * ± 11.2
	3	48.3 ± 12.3	28.1 ± 21.0	88.3 * ± 13.7	86.7 * ± 13.1	88.3 * ± 13.7	88.3 * ± 17.7	95.0 * ± 8.1
	4	53.3 ± 17.2	48.3 ± 22.8	93.3 * ± 16.1	90.0 * ± 16.1	85.0 * ± 20.0	90.0 * ± 17.9	90.0 * ± 14.1
	24	40.0 ± 17.9	48.3 ± 14.6	71.7 * ± 23.6	83.3 * ± 13.6	68.3 * ± 24.2	71.7 * ± 29.4	73.3 * ± 29.6
	48	43.3 ± 11.7	55.0 ± 20.9	76.7 * ± 28.5	65.0 * ± 16.6	55.0 ± 15.8	68.3 * ± 34.6	63.3 * ± 36.7
	72	45.0 ± 19.3	51.7 ± 20.0	71.7 * ± 11.2	61.7 ± 19.3	48.3 ± 25.4	73.3 * ± 23.8	76.7 * ± 19.6
	96	56.7 ± 26.3	56.7 ± 17.9	57.0 ± 17.1	66.7 * ± 20.8	43.3 ± 21.1	66.7 * ± 33.3	71.7 * ± 17.7
	168	42.8 ± 29.4	53.3 ± 29.2	51.7 ± 21.4	55.0 ± 22.3	60.0 ± 29.6	70.0 * ± 21.9	63.3 * ± 18.9
Mean repellency (%) ± standard deviation		49.3 ± 4.2	47.5 ± 6.3	77.5 ± 3.7	78.9 ± 9.6	74.1 ± 3.9	82.9 ± 3.4	85.4 ± 2.4

* Repellency was considered significant when the number of ticks on the untreated side of the Petri dish was higher than that observed on the treated side, with a statistically significant difference at the 5% level, according to the chi-square test. Control (ethanol): Petri dish containing one semicircle of filter paper treated with 50% ethanol and one untreated semicircle. Control (blank): Petri dish containing two semicircles of untreated filter paper, with no compounds applied.

**Table 3:** Repellency percentage (mean ± standard deviation) of unfed *Amblyomma sculptum* nymphs exposed to 7% DEET and eugenol (1% and 2%), either alone or in combination, in a Petri dish assay conducted under laboratory conditions (27 ± 1 °C and 70 ± 10% relative humidity).

Evaluation Time (Minutes)		Control (Ethanol)	Control (Blank)	1% Eugenol	2% Eugenol	7% Deet	7% Deet + 1% Eugenol	7% Deet + 2% Eugenol
Minutes	1	53.3 ± 18.9	53.3 ± 25.8	73.3 * ± 22.5	85.0 * ± 14.6	83.3 * ± 17.6	98.3 * ± 5.3	93.3 * ± 8.6
	15	60.0 ± 19.6	36.7 ± 15.3	68.3 * ± 16.6	88.3 * ± 13.7	85.0 * ± 18.3	86.7 * ± 18.9	90.0 * ± 11.7
	30	50.0 ± 24.8	41.7 ± 22.6	76.7 * ± 14.1	93.3 * ± 11.7	91.7 * ± 14.2	91.7 * ± 11.8	85.0 * ± 14.6
Hours	1	41.7 ± 21.2	55.0 ± 13.7	86.7 * ± 15.3	91.7 * ± 11.8	91.7 * ± 11.8	91.7 * ± 8.8	83.3 * ± 22.2
	2	55.0 ± 20.9	46.7 ± 13.1	91.7 * ± 14.2	88.3 * ± 13.7	89.7 * ± 14.3	93.3 * ± 8.6	88.3 * ± 13.7
	3	50.0 ± 11.1	33.1 ± 19.5	93.3 * ± 11.7	91.7 * ± 8.8	88.3 * ± 13.7	93.3 * ± 8.6	86.7 * ± 15.3
	4	50.0 ± 22.2	60.0 ± 14.1	85.0 * ± 18.3	81.7 * ± 21.4	85.0 * ± 20.0	95.0 * ± 8.1	88.3 * ± 15.8
	24	38.3 ± 23.6	41.7 ± 19.6	79.2 * ± 18.9	81.7 * ± 16.6	68.3 * ± 24.2	85.0 * ± 25.4	88.3 * ± 15.8
	48	41.7 ± 11.8	48.3 ± 20.0	68.8 * ± 9.2	81.7 * ± 18.3	55.0 ± 15.8	85.0 * ± 25.4	81.7 * ± 14.6
	72	43.3 ± 11.7	46.7 ± 13.1	70.0 * ± 18.9	77.5 * ± 15.7	48.3 ± 25.4	78.3 * ± 23.6	83.3 * ± 19.2
	96	46.7 ± 27.0	56.7 ± 16.1	62.2 ± 22.9	58.3 ± 23.9	43.3 ± 21.1	80.0 * ± 24.6	76.7 * ± 16.1
	168	41.2 ± 28.4	46.7 ± 13.1	74.2 * ± 31.0	75.8 * ± 21.0	60.0 ± 29.6	78.3 * ± 22.3	81.0 * ± 15.5
Mean repellency (%) ± standard deviation		47.6 ± 6.8	47.2 ± 7.7	77.6 ± 9.6	82.9 ± 3.2	74.1 ± 3.9	88.1 ± 4.2	85.5 ± 4.0

* Repellency was considered significant when the number of ticks on the untreated side of the Petri dish was higher than that observed on the treated side, with a statistically significant difference at the 5% level, according to the chi-square test. Control (ethanol): Petri dish containing one semicircle of filter paper treated with 50% ethanol and one untreated semicircle. Control (blank): Petri dish containing two semicircles of untreated filter paper, with no compounds applied.

## Data Availability

All subjects gave their informed consent for inclusion before they participated in this study. This study was conducted in accordance with the Declaration of Helsinki, and the protocol was approved by the Ethics Committee of no. 4.955.565.

## Abbreviations

The following abbreviations are used in this manuscript:

ANOVA	Analysis of variance
BSF	Brazilian spotted fever
CEP	Research ethics committee (Comitê de Ética em Pesquisa)
CEUA	Animal use ethics committee (Comissão de Ética no Uso de Animais)
DEET	N, N-diethyl-3-methylbenzamide
EO(s)	Essential oil(s)
EVZ	School of veterinary medicine and animal science (Escola de Veterinária e Zootecnia)
PAHO	Pan American Health Organization
PPE	Personal protective equipment
RH	Relative humidity
SBP	Brazilian society of parasitology (Sociedade Brasileira de Parasitologia)
SD	Standard deviation
UFG	Federal University of Goiás
